# Long-Term Effects of Single-Dose Cephalosporin or Macrolide Use on the Prevalence of AmpC and Extended-Spectrum β-Lactamase Producing *Escherichia coli* in the Feces of Beef Cattle

**DOI:** 10.3390/microorganisms10102071

**Published:** 2022-10-20

**Authors:** Gizem Levent, Ashlynn Schlochtermeier, Javier Vinasco, Jenny Jennings, John Richeson, Samuel E. Ives, Keri N. Norman, Sara D. Lawhon, Guy H. Loneragan, H. Morgan Scott

**Affiliations:** 1 Department of Veterinary Pathobiology, Texas A&M University, College Station, TX 77845, USA; 2 School of Veterinary Medicine, Texas Tech University, Amarillo, TX 79106, USA; 3 Department of Agricultural Sciences, West Texas A&M University, Canyon, TX 79016, USA; 4 Department of Veterinary Integrative Biosciences, Texas A&M University, College Station, TX 77845, USA

**Keywords:** *Escherichia coli*, ESBL, AmpC, feedlot cattle

## Abstract

Extended-spectrum-β-lactamase (ESBL) and AmpC-lactamase-producing Enterobacteriaceae are serious public health threats. Due to an increasing number of reports of ESBL and AmpC producing *Escherichia coli* in agricultural settings, it is critical to understand the relationship between the use of two of the highest priority critically important human antibiotics (e.g., third generation cephalosporins [3GC] and macrolides) in food animals and their potential contribution to the selection of ESBL/AmpC *E. coli*. The objective of our randomized controlled feedlot trial was to measure the effects of ceftiofur crystalline-free acid and tulathromycin on 3GC resistant fecal *E. coli* populations in cattle before and at various time points after treatment up to and including at slaughter. Multi-level mixed-effects linear regression showed no effect of ceftiofur and tulathromycin on 3GC *E. coli* CFU counts at slaughter (Day 99); however, a significant (*p* < 0.05) population shift was observed from susceptible to 3GC resistant *E. coli* immediately after ceftiofur administration (Day 7). Among 799 fecal samples screened using selective media, 17.7% were ESBL/AmpC *E. coli* positive, which were further tested for phenotypic antibiotic susceptibility. The majority of the isolates from these plates were multidrug-resistant (94.3%) and expressed either AmpC (78.1%) or ESBL (28.1%) phenotype. A subset of isolates was whole-genome sequenced (n = 20) and identified to harbor chromosomal and/or plasmidal *bla* genes such as CMY-2, CTX-M, and TEM. Our findings show a time-dependent selection of antibiotics on 3GC-resistant *E. coli*. High prevalence of multidrug-resistant ESBL/AmpC *E. coli* found in cattle feces highlights the importance of prudent use of antibiotics in livestock.

## 1. Introduction

Since they were first approved in the 1950s, the use of antibiotics in food-producing animals has been the subject of ongoing debate and represents a global public health concern due to the rise of antibiotic-resistant (AMR) foodborne pathogens [1,2,3]. Depending on the dose, formulation, route of administration, class, and duration of use, it has been shown that administration of antibiotics can select for AMR pathogens in the intestinal microbiota of cattle, which may result in contamination of final food products at slaughter [4,5]. Consumption of contaminated and under-cooked food products (e.g., beef, pork, eggs, or chicken) can result in exposure to multidrug-resistant (MDR) foodborne pathogens leading to gastrointestinal infections in humans [6]. Within the last few decades, foodborne pathogens (e.g., *Salmonella enterica* subspecies *enterica* non-Typhi and *Campylobacter* spp.) that are resistant (or have reduced susceptibility) to cephalosporins (e.g., ceftriaxone), macrolides (e.g., azithromycin), or fluoroquinolones (e.g., ciprofloxacin)—which are all highest priority critically important antibiotics for the treatment of severe invasive gastrointestinal infections in human [7]—have been a great public health concern due to their increasing prevalence and distribution among humans, food products, and livestock [8,9].

*Escherichia coli* is a member of the Enterobacteriaceae family, and except for a relatively small group of human pathogenic and toxin-producing serotypes, is also considered a commensal member of the intestinal microbiota of animals and humans. In general, the AMR profile of non-type-specific *E. coli* in food products is considered an indicator of other Gram-negative AMR pathogens that are important for food safety. Cephalosporin-, and fluoroquinolone-resistant bacteria emerged in livestock *E. coli* within a decade after the introduction of these antibiotics for veterinary medicine [1]. More recently, reduced susceptibility to macrolides has surprisingly appeared in Enterobacteriaceae, perhaps following on the heels of the use of azithromycin for *S*. Typhi in humans.

Cephalosporins are a major subclass of β-lactam antibiotics and their spectrum of activity and use has gradually been extended against Gram-negative pathogens over the last few decades [10]. Lower-generation cephalosporins (e.g., 1st and 2nd generation) are important human antibiotics that are mainly used for surgical prophylaxis or treatment of uncomplicated community-acquired infections that caused by non-AMR pathogens. Higher-generation cephalosporins (e.g., 3rd generation [3GC] and above, referred to as extended-spectrum β-lactams) are considered highest priority critically important human antibiotics due to their broad-spectrum bactericidal activity against severe community-acquired infections caused by Gram-negative pathogens [10]. Unlike lower-generation cephalosporins, higher-generation cephalosporins can also penetrate the blood–brain barrier, which makes them a crucial antibiotic for bacterial meningitis [10,11].

According to the Centers for Disease Control and Prevention (CDC), Enterobacteriaceae resistant to extended-spectrum β-lactams are considered a serious public health threat [8,9]. In these microorganisms, resistance against lower- and higher-generation β-lactams is maintained by various, mostly acquired, β-lactamase encoding *bla* genes such as CMY, CTX-M, TEM, and SHV [12]. These *bla* genes are usually located on bacterial plasmids that can be horizontally transferred between and within bacterial species [13,14]. Transfer of these genes into the bacterial chromosome can also occur via mobile genetic elements [15]. Therefore, plasmids and mobile genetic elements are known to play a major role in the rapid dissemination of ESBL/AmpC resistance among Enterobacteriaceae—including foodborne pathogens—found in humans, livestock, the environment, and food products [12,16,17,18].

Ceftiofur is the only 3GC that has been introduced for use in veterinary medicine; this occurred in the late 1980s in the United States. Since then, it has become one of the most effective antibiotics used for bacterial infectious diseases in animals including food-producing animals such as dairy and beef cattle, poultry, and swine in the United States. In cattle, ceftiofur crystalline-free acid administered at the base of the pinna (6.6 mg/kg body weight) is currently used for the treatment and control of bovine respiratory disease (BRD) in beef cattle. Within a decade after the approval of ceftiofur for cattle use, ESBL/AmpC-producing *E. coli* and *Salmonella* were reported in cattle feces and beef products [16,17,18,19,20]. Later, high levels of *Salmonella* Heidelberg with 3GC resistance in broiler chickens was attributed to *in ovo* injections of ceftiofur in hatcheries. This resulted in the prohibition of most extra-label uses of ceftiofur in food animals in 2012 [21]. However, despite the restricted use of cephalosporins in animals, according to the Annual Domestic Sales and Distribution Report of 2019 published by the United States Food and Drug Administration (FDA), since 2010, the estimated annual kg of cephalosporins sold for animal use has increased from 24,588 kg to 29,830 kg in 2019 with the majority being sold for cattle use in the United States [22].

Bovine respiratory disease (BRD) is the most common infectious cattle disease affecting more than 96% of cattle feedlots with >1000 head capacity in the United States [23]. Among these feedlots, approximately 16% of cattle develop BRD and almost all are treated with antibiotics. To control this disease, antibiotics may be administered as metaphylaxis to groups of animals in addition to treatments given to individual sick animals. Ceftiofur crystalline-free acid (CCFA) is the only long duration extended-spectrum cephalosporin antibiotic that is approved for the management of BRD in cattle herds [23]. Its usefulness is in large part due to its long-acting formulation and single-dose effectiveness to reduce BRD-related mortality and morbidity rates in cattle feedlots [24,25].

In the literature, numerous studies have assessed the potential short-term (<28 days) effects of ceftiofur on the ESBL/AmpC-producing *E. coli* populations or fecal microbiota of beef cattle [26,27,28,29,30]. However, these studies were not sufficient to reveal the potential effects of ceftiofur on ESBL/AmpC-producing *E. coli* at slaughter age; that is, after a 3- to 9-month feeding period. Besides the potential selection of cephalosporins on ESBL/AmpC *E. coli*, the use of other classes of antibiotics may result in the co-selection of ESBL/AmpC *E. coli* due to the potential co-existence of AMR encoding genes in the bacterial population. Similar to ceftiofur, tulathromycin (an analog of azithromycin) is in the macrolide class and is also commonly used in BRD management in U.S. cattle feedlots. Tulathromycin given subcutaneously in the neck (2.5 mg/kg body weight) also is indicated for the treatment and control of BRD in beef cattle. Macrolides are also highest priority critically important antibiotics for human medicine. Emerging macrolide resistance (actually, reduced susceptibility to macrolides), especially resistance to azithromycin in Enterobacteriaceae, is also an emerging public health concern [9].

Currently, the use of these antibiotic classes in food-producing animals is not recommended by the World Health Organization (WHO). This recommendation was built upon the currently available research suggesting a parallel relationship between the use of critically important human antibiotics in food animals and emerging resistance in Enterobacteriaceae; however, such recommendations regarding the use of these antibiotics was left conditional due to the lack of high-quality, peer-reviewed evidence [11,31].

To fill the forementioned research gaps, we designed a randomized controlled field study to understand the temporal dynamics of fecal ESBL/AmpC-producing *E. coli* prevalence and quantity in cattle following a single-dose of ceftiofur or tulathromycin followed from the time of treatment, through the feeding period, and on until until slaughter-ready age. Our primary focus was to measure the distribution and quantity of 3GC susceptible versus resistant *E. coli* in cattle feces interacting by sampling day and antibiotic treatment. In addition, ESBL/AmpC-producing *E. coli* were further isolated using selective media, and their phenotypic antimicrobial susceptibility profiles were investigated using a broth microdilution method. Finally, a subset of these isolates was whole-genome sequenced to identify the sequence types, AMR genes and point mutations, and plasmids, along with the origin of the β-lactam resistance and related mobile genetic elements.

## 2. Materials and Methods

The West Texas A&M University (WTAMU)/Cooperative Research, Educational, and Extension Team Institutional Animal Care and Use Committee (protocol no. 05-09-15) approved this animal field trial, while the microbiological assays were carried out under the supervision of the Texas A&M University Institutional Biosafety Committee (IBC 2017-049).

### 2.1. Study Design

A total of 134 crossbreed yearling healthy and backgrounded beef cattle purchased from two different sources in the Texas high plains, USA were transported to WTAMU Research Feedlot located in Canyon, TX, USA. After a 3-day acclimation period, cattle were source- and weight-blocked into four groups. Cattle in each block were randomly assigned into 3 treatment pens consisting of 11–12 cattle per pen. Cattle in each treatment pen were injected with a single dose of either tulathromycin (Draxxin^®^, Zoetis, Kalamazoo, MI, USA, at 2.5 mg/kg, subcutaneously in the neck), ceftiofur crystalline-free acid (Excede^®^, Zoetis, Kalamazoo, MI, USA, at 6.6 mg/kg, subcutaneously in the posterior aspect of the pinna of the ear) per label instructions on the first day of the study (referred as Day 0), or else remained as a control without any antibiotic treatment. One or two cattle in each antibiotic treatment pen also remained as sentinel controls and received no treatment.

Individual cattle identifiers were given to each steer and laboratory and feedlot personnel were double-blind to the treatment information of individual steers during the entire study. Cattle were fed diets that met market requirements for beef finishing. Throughout the feeding season, the diet consisted primarily of wet corn gluten, chopped corn stalks, corn oil, steamed-flaked corn, and mineral supplements (all of which were free of antibiotics). In each pen, there were two automatic watering bowls. Any animal that required additional antibiotic treatment because of illness was excluded from the study and removed from its original pen.

A 25 g fecal sample was collected per rectum from each steer using sterile obstetric gloves before the administration of antibiotics at the onset of the study (referred to as Day 0). After the collection of the first fecal sample, antibiotics were administered and fecal samples were repeatedly collected on Days 7, 14, 28, 56, and 99. After Day 99, cattle were transported to slaughter.

After each sample collection day, samples were shipped overnight at 4 °C to the laboratory at Texas A&M University in College Station, TX. Each sample was homogenized with glycerol (1:1 ratio) upon arrival in the laboratory, preserved in cryo-vials, and stored at −80 °C for further microbiological analyses.

### 2.2. Bacterial Quantification and Confirmation

Bacterial quantification and confirmation were performed similar to the published method by Taylor et al., 2019 [32]. Briefly, frozen fecal samples were initially thawed, and 0.5 g of fecal/glycerol matter was weighed from each sample and transferred into a 5 mL sterile vial with 4.5 mL sterile 1X phosphate-buffered saline (PBS) (Gibco, Thermo Fisher Scientific, Gaithersburg, MD, USA) and homogenized using a vortex. Later, 50 µL of the suspension was spiral plated onto plain MacConkey agar (MAC) plates (BD Difco, Franklin Lakes, NJ, USA) and 3GC selective MacConkey agar plates containing 4 µg/mL ceftriaxone (MCef) (Sigma-Aldrich, St. Louis, MO, USA) using the Eddy Jet 2 (Neutec Group Inc., Farmingdale, NY, USA). Plates were incubated at 37 °C for 18 to 24 h and presumptive *E. coli* colonies that grew on MAC and MCef plates (3GC-resistant *E. coli*) were counted using an automated Flash & Go instrument (Neutec Group Inc., Farmingdale, NY, USA). For biochemical confirmation of *E. coli* colonies that grew on MCef plates, one colony from each plate was randomly picked and streaked on tryptic soy agar with 5% sheep blood (Becton, Dickinson, Franklin Lakes, NJ, USA), and incubated for 18 h at 37 °C. After incubation, a single colony from each plate was indole tested using James Reagent (bioMérieux, Marcy-l’E’toile, France).

### 2.3. Detection of ESBL/AmpC Producing E. coli

In parallel to MAC and MCef plating, a 0.5 mL suspension was mixed with 4.5 mL of MacConkey broth (BD Difco, Franklin Lakes, NJ, USA) with 2 µg /mL ceftriaxone and incubated overnight at 37 °C for 18–24 h for selective enrichment. A 50 µL aliquot of pre-enriched suspension was spiral plated on CHROM agar ESBL (extended-spectrum β-lactamases) plates (HardyCHROM™ ESBL, Hardy Diagnostics, CA, USA). Plates were incubated at 37 °C for 18–24 h. One presumptive ESBL/AmpC *E. coli* colony expressing dark pink to reddish phenotype on an CHROM agar ESBL plate was randomly selected and streaked on a tryptic soy agar with 5% sheep blood plate (Becton, Dickinson, Franklin Lakes, NJ, USA) and incubated for 18 h at 37 °C. One colony from each plate was further selected and identified using matrix-assisted laser desorption ionization-time of flight mass spectrometry (MALDI-TOF MS, Bruker Daltonics GmbH, Leipzig, Germany). Confirmed *E. coli* isolates were further tested for phenotypic antibiotic susceptibility. This method was also published by Taylor et al., 2019 [32].

#### 2.3.1. Phenotypic Antibiotic Susceptibility

Phenotypic antibiotic susceptibility profiles of MALDI-TOF confirmed *E. coli* isolates were determined via broth microdilution using the TREK Sensititre system™ (Thermo Scientific Microbiology, Oakwood Village, OH, USA) and Gram-negative NARMS plate (CMV3AGNF, Thermo Fisher Scientific, Waltham, MA, USA). Minimum inhibitory concentrations (MIC) of 14 antibiotics (amoxicillin-clavulanic acid, ampicillin, azithromycin, cefoxitin, ceftiofur, ceftriaxone, chloramphenicol, ciprofloxacin, gentamicin, nalidixic acid, streptomycin, sulfisoxazole, tetracycline, and trimethoprim-sulfamethoxazole) were determined for each isolate using NARMS, Manual of Laboratory Methods protocol for Antimicrobial Susceptibility Testing of *E. coli* (Manual of Laboratory Methods, The National Antimicrobial Resistance Monitoring System. Available online: https://www.fda.gov/media/101423/download accessed on 1 October 2021). *E. coli* ATCC (American Type Culture Collection) 25922, *E. coli* ATCC 35218, *Pseudomonas aeruginosa* ATCC 27853, *Staphylococcus aureus* ATCC 29213, and *Enterococcus faecalis* ATCC 29212 strains were also included for quality-control. Results were interpreted using the breakpoints listed by the Clinical and Laboratory Standards Institute (CLSI) to classify phenotypic antibiotic resistance profiles of the isolates as either susceptible, intermediate, or resistant. MIC breakpoint criteria for azithromycin and streptomycin have not been established for *E. coli* by the CLSI; therefore, the National Antimicrobial Resistance Monitoring System (NARMS) established breakpoints were used for azithromycin and streptomycin (https://www.cdc.gov/narms/antibiotics-tested.html [accessed on 1 April 2021]). Isolates resistant to 3 or more antibiotic classes were classified as multidrug-resistant (MDR). A selection of presumptive ESBL/AmpC isolates obtained from different treatments, pens, and days were further subjected to whole-genome sequencing (WGS) for genotypic characterization.

#### 2.3.2. Whole-Genome Sequencing and Bioinformatics

Bacterial DNA from selected isolates was extracted using the QIAamp 96 DNA QIAcube HT kit and QIAcube HT platform (Qiagen, Valencia, CA, USA) according to the manufacturer’s instructions. DNA purity was assessed at a 260/280 nm ratio of absorbance and quantity was evaluated by the Quant-IT PicoGreen double-strand DNA (dsDNA) assay kit (Thermo Fisher Scientific, Waltham, MA, USA) using a FLUOstar Omega multimode microplate reader (BMG Labtech, Cary, NC, USA) following the manufacturer’s instructions. Sequencing libraries were generated using the Illumina Nextera XT kit (Illumina, San Diego, CA, USA) and sequenced using the Illumina MiSeq reagent v3 (600 cycles, 300 paired end reads) chemistry kit on the MiSeq platform (Illumina, San Diego, CA, USA).

Nextera XT adapters and reads that were below the Phred quality score of 33 were trimmed using Trimmomatic v.0.36 [33]. Trimmed reads were assembled using SPAdes v.3.11.1 [34]. Assemblies were further assessed to determine the corresponding 7-gene multi-locus sequence types (MLST) using mlst_check v2.1.1 tool [35] and *E. coli* MLST database [36]. Acquired AMR gene and plasmid contents were determined using ABRicate v.0.8.7 (Available online: https://github.com/tseemann/abricate accessed on 1 May 2021) along with ResFinder [37] and PlasmidFinder [38] databases. In addition to the acquired AMR genes, chromosomal point mutations were also screened using PointFinder [39] on the Center for Genomic Epidemiology (CGE) platform (Available online: https://www.genomicepidemiology.org/ accessed on 1 May 2021). Contigs carrying β-lactamase genes were extracted and screened for mobile genetic elements using MobileElementFinder [40] on CGE and the origin of the contigs was predicted using mlplasmids v.1.0.0 (Available online: https://sarredondo.shinyapps.io/mlplasmids/ accessed on 1 May 2021) [41].

### 2.4. Statistical Analysis

Statistical analyses were performed using Stata v.16.1. Both MAC and MCef *E. coli* CFU counts (per gram fecal samples) were obtained and log_10_ transformed for downstream statistical analyses. Transformed log_10_ count data were examined and tabulated by day and treatment. The proportion of 3GC (ceftriaxone) resistant *E. coli* was calculated by taking the anti-log of the log_10_ CFU difference of MCef vs. MAC counts (log_10_ MCef CFU–log_10_ MAC CFU). To assess treatment effects on the total *E. coli* population and 3GC-resistant *E. coli* proportion, multi-level mixed-effects linear regression models were performed with sampling day and treatment as fixed effects, and the interaction term of day and treatment since the design of the study was two by two full-factorial. Pen and individual animal identifiers were also forced into the models as random effects to account for potential clustering effects. Day 0 and control (no treatment) groups were considered as baseline values for period and antibiotic, respectively. From each model, marginal linear predictions and their 95% confidence intervals by day and treatment were estimated and estimates were plotted. The contrast of average marginal effects of model parameters (fixed effects and interaction terms) was identified by adjusting according to Bonferroni’s method to obtain significance levels.

The effect of treatments on ESBL/AmpC producer *E. coli* prevalence was examined using a multi-level mixed-effect logistic regression accounting for the same model parameters included in the linear regression model. Isolates that were phenotypically resistant to ceftiofur and ceftriaxone but susceptible to cefoxitin were considered ESBL, whereas the isolates that were resistant to amoxicillin-clavulanic acid and cefoxitin along with resistant to ceftiofur and ceftriaxone were considered AmpC phenotype. Prevalence of presumptive ESBL/AmpC producing *E. coli*, along with the *E. coli* isolates that showed ESBL phenotype and AmpC phenotype in feces were tabulated by day and treatment.

## 3. Results

Among 134 study cattle, two study cattle were lost to follow-up after Day 28 because of reported illness; therefore, 132 cattle completed the study. A total of 799 fecal samples were collected from the beginning (Day 0) to the end of the study (Day 99).

### 3.1. Count Data

A total of 761 MAC plates (95.2%) and 100 MCef plates yielded countable colonies with overall log_10_ CFU means of 6.11; 95%CI: 6.05–6.17, and 4.6; 95%CI: 4.51–4.8, respectively. Among MAC plates from which CFU counts were obtained, mean log_10_ CFU counts were 6.10; 95%CI: 5.97–6.02, 6.22; 95%CI: 6.11–6.33, and 6.03; 95%CI: 5.90–6.13 for ceftiofur, tulathromycin, and control cattle, respectively. Among MCef plates from which CFU counts were obtained, mean log_10_ CFU counts were 4.64; 95%CI: 4.36–4.96, 4.71; 95%CI: 4.36–5.06, and 4.75; 95%CI: 4.41–5.09 for ceftiofur, tulathromycin, and control cattle, respectively. Mean, standard deviation, minimum and maximum log_10_ transferred CFU counts obtained from samples by day and individual treatment are presented in Table S1.

**Figure 1:**
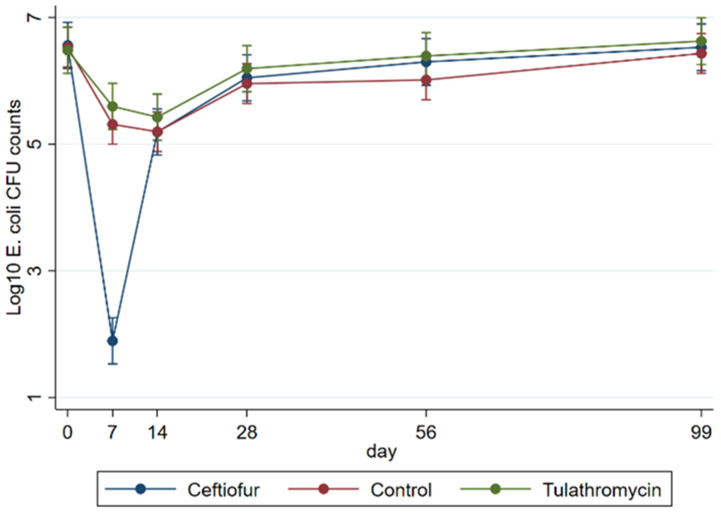
Modeled marginal linear predictions of treatment effects on total Log_10_ CFU *E. coli* recovered from fecal samples before (Day 0) and after (Days 7–99) treatment.

Linear regression results obtained from multi-level mixed-effects models showed that on Day 7, ceftiofur treatment resulted in a significant reduction (*p*= 0.022) in log_10_ CFU MAC counts compared to the control and tulathromycin groups (ceftiofur: 1.82; 95% CI:1.08–2.5, control: 5.45 log_10_ CFU; 95% CI:5.08–5.81, and tulathromycin: 5.47: 95%CI: 5.04–5.90). Overall, there was a significant log_10_ CFU reduction (*p* = 0.000) observed in ceftiofur-treated animals on Days 7 and 14 (1.82; 95% CI:1.08–2.5, and 5.12; 95%CI: 4.6–5.6) when compared to the baseline value (6.4 log_10_ CFU: 95% CI: 6.09–6.89). However, after Day 14, the counts recovered back to the baseline values and were not significantly different from the other treatment groups (*p* >0.05) (Figure 1).

**Figure 2:**
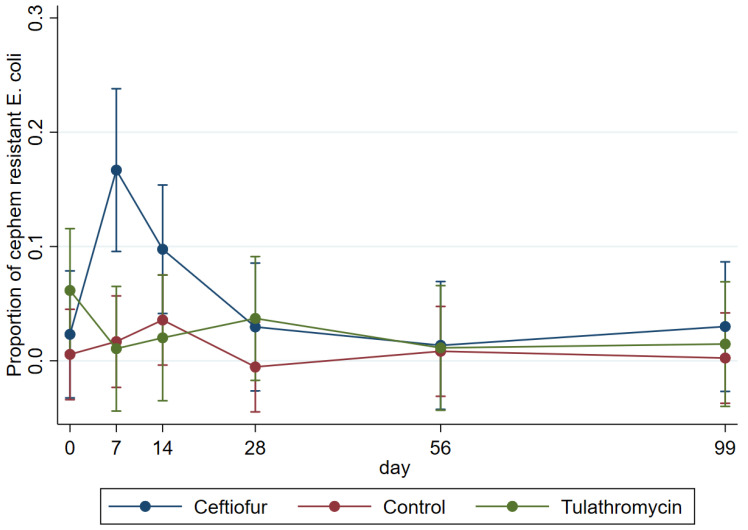
Modeled marginal proportions (i.e., antilog of log_10_ difference between MAC and MCef) of 3GC-resistant *E. coli* over the total *E. coli* population recovered from fecal samples before (Day 0) and after (Days 7–99) treatment.

Model estimates for the proportion of the 3GC-resistant *E. coli* population showed that there was no significant effect of day and treatment observed up to the slaughter age (*p* > 0.13); however, the proportion of resistant *E. coli* was significantly (*p* = 0.029) higher on day 7 (0.16; 95%CI: 0.09–0.23) in ceftiofur treated cattle when compared to control (0.01; 95%CI: −0.23 −0.05) and tulathromycin (0.01; 95%CI: −0.43 −0.06) treated animals (Figure 2).

### 3.2. ESBL/AmpC E. coli Populations

**Figure 3:**
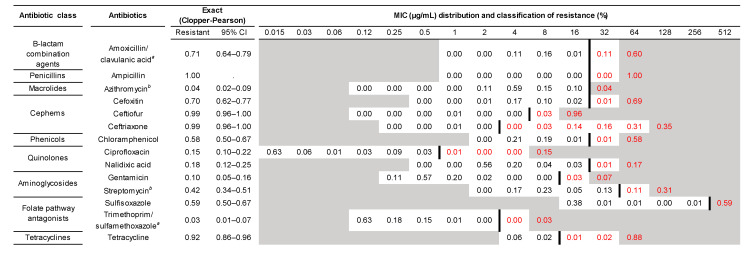
Proportions and 95% confidence intervals of bacterial phenotypic resistance and corresponding MIC distributions for 142 ESBL or AmpC *E. coli* isolates tested against 14 antibiotics. The shaded areas represent the out-of-dilution ranges for antibiotics on the plate. Therefore, numbers in the shaded areas represent right-censored MIC resistant values. The proportion of resistant isolates is indicated in red, and breakpoints for resistant classification are shown as bolded lines between the black and red numbers. ^a^ MIC of the first antibiotic in a combination is listed. ^b^ NARMS consensus breakpoints were used for the classification of resistance.

Among 799 samples, 142 isolates were recovered from CHROM agar ESBL plates, confirmed by MALDI-TOF, and classified as either ESBL or AmpC *E. coli*. All ESBL/AmpC isolates were tested for phenotypic antibiotic susceptibility. Among the 142 isolates, 101 isolates (71.1%; 95%CI: 62.9–78.4) exhibited an AmpC phenotype, whereas 40 isolates (28.1%; 95%CI: 20.9–36.3) exhibited an ESBL phenotype. One isolate remained unclassified due to its complex phenotypic antibiotic susceptibility pattern. Phenotypic antibiotic susceptibility results showed that all isolates were resistant to at least 3 antibiotics tested, including to ampicillin. The vast majority of all isolates were MDR (94.3%), which meant they were resistant to three or more antibiotic classes. Among these, three isolates were resistant to all nine classes of antibiotics tested. The most common MDR profile of the isolates (n = 74) was amoxicillin-clavulanic acid, ampicillin, cefoxitin, ceftiofur, ceftriaxone, and tetracycline. A subset of these isolates (n = 23) was also additionally resistant to chloramphenicol, streptomycin, and sulfisoxazole, and the remaining 11 isolates also expressed resistance against gentamicin. Resistance to 3rd generation cephalosporins (ceftriaxone and ceftiofur) was highly prevalent and observed in 141 of the 142 isolates. In addition, cefoxitin resistance was observed in 69.7% (n = 99) of the isolates tested. Furthermore, 17.6% (25/142) of the isolates were resistant to fluoroquinolones and quinolones (e.g., ciprofloxacin and/or nalidixic acid) and an additional 14.7% (n = 21) of the isolates showed reduced susceptibility to ciprofloxacin (MIC of 0.12–1 μg/mL). Phenotypic macrolide resistance (e.g., azithromycin) was only observed in six isolates. MIC distributions of the isolates, along with the corresponding MIC breakpoints for resistance classification against the 14 antibiotics tested in this study are shown in the ‘squashtogram’ in Figure 3.

On Day 0, before the antibiotic treatment, ESBL/AmpC producing fecal *E. coli* prevalence was non-significantly (*p* > 0.05) highest (27.5%; 95%CI:14.6–43.8) in the cattle group that received tulathromycin (n = 40), and lowest (10.0%; 95%CI: 2.7–23.6) in the ceftiofur treated group (n = 40). Before treatment, 13.2% (95%CI: 5.4–25.3) of the control cattle (n = 53) were ESBL/AmpC-producing *E. coli* positive. However, one week after treatment, on Day 7, ceftiofur-treated cattle reached the highest prevalence (27.5%; 95%CI: 14.6–43.8), compared to the tulathromycin (15.0%; 95%CI: 5.7–29.8) and control (5.5%; 95%CI: 1.1–15.3) groups. Upon finishing the feeding period, just before slaughter (on Day 99), ESBL/AmpC producing fecal *E. coli* population dynamics had recovered back to the initial levels with the highest recorded prevalence in the tulathromycin-treated cattle (20.5%; 95%CI: 9.2–36.4), followed by control (18.5%; 95%CI: 9.2–31.4), and ceftiofur treated cattle (17.9%; 95%CI: 7.2–33.5); importantly, none of these differed significantly (*p* > 0.05) with overlapping 95% confidence intervals.

In parallel, after the classification of ESBL producers (based on phenotypic susceptibility testing), before treatment results showed that ESBL-producing *E. coli* prevalence values were 7.5% (3/40) and 9.4% (5/53) for tulathromycin and control groups, respectively. There were no ESBL *E. coli* isolated from the 40 cattle receiving ceftiofur on Day 0. However, on Day 7 ESBL *E. coli* prevalence in ceftiofur-treated cattle increased and reached 15.0% (6/40) and was higher than the tulathromycin (2.5%; 1/40), and control (3.7%; 2/54) cattle. Right before slaughter, at the end of the study, there were no ESBL *E. coli* isolated from ceftiofur-treated cattle, whereas only one steer from each of the tulathromycin and control group were positive for ESBL *E. coli*. The proportion of ESBL/AmpC, and only ESBL, and AmpC-producing *E. coli* isolates from cattle by day and treatment are presented in Table 1.

### 3.3. Genomic Characterization of AmpC/ESBL Producing E. coli

A subset of AmpC (n = 7) and ESBL (n = 13) phenotypic *E. coli* isolates recovered from the combination of various day, pen, and individual treatment groups was selected and further subjected to whole-genome sequencing for genotypic characterization. Based on the detection of the AMR genes and chromosomal point mutations, plasmids, mobile genetic elements, and contig origin predictions, 7-gene MLST ST224 was the most common ESBL *E. coli* sequence type (n = 4); all of which carried a chromosomally encoded ESBL gene *bla*_CTX-M-32_ along with the insertion sequence ISKpn26, and another *bla*_TEM-1A_ gene was also found along with insertion sequence ISVsa3 which was predicted to be plasmidal (Table S2). Besides the *bla* gene carrying contigs, ST244 isolates were MDR and harbored additional AMR elements and chromosomal point mutations such as *flo*R, *gyr*A, *par*C, *par*E, *sul*2, *tet*(A), *aph*(3″)-Ib, and *aph*(6)-Id. Similar to ST224, two isolates of ST8443 also harbored the same plasmidal *bla*_TEM-1A_ and ISVsa3 element; however, a *bla*_CTX-M-1_ encoding plasmidal contig was found in this group which also carried the macrolide-resistance gene *mph*(A), though as expected it was not phenotypically resistant to azithromycin (reduced susceptibility at MIC: 16 µg/mL). Similarly, a contig carrying both the *bla*_CTX-M-1_ and *mph*(A) genes was also observed in two isolates (ST306) that were also not phenotypically resistant to azithromycin (MIC:16 µg/mL). Although both ST306 isolates harbored other identical AMR genes, one of these isolates was classified as AmpC due to additional phenotypic amoxicillin-clavulanic acid and cefoxitin resistance being observed. Two ESBL isolates were identified as ST17 and harbored a contig carrying *bla*_CTX-M-27_ and an IS102 element. ST17 isolates were phenotypically resistant to ampicillin, ceftiofur, and ceftriaxone and did not harbor additional AMR genes. The remaining ESBL isolates were found to be variable and single STs and harbored plasmidal or chromosomally encoded *bla*_CTX-M_ genes such as *bla*_CTX-M-1_ or a *bla*_CTX-M-55_ located in IncFIC(FII)_1 plasmids. One presumptive single azithromycin-resistant (MIC > 16 μg/mL) ESBL isolate (ST744) that was sequenced was also carrying the *mph*(A) gene. Because the highest concentration of azithromycin on the plate was 16 μg/mL and the clinical breakpoint is 32 μg/mL, it is not possible to state with certainty that this isolate was actually resistant. Interestingly, this isolate harbored a chromosomal *bla*_CTX-M-1_ gene that was not located in the same contig as the *mph*(A) gene (Table S2).

Six of the seven AmpC isolates harbored *bla*_CMY-2_ genes. One of the most common STs for AmpC *E. coli* was ST156 (n = 3), which was recovered from a single ceftiofur-treated animal (ID:4243) both before (Day 0) and after the treatment on Days 7 and 28. These isolates were MDR, and all isolates carried a *bla*_CMY-2_ gene and an ISEc9a element located on an IncX4_2 plasmid. Besides the plasmidal *bla*_CMY-2_ gene, these isolates also harbored a chromosomally encoded *bla*_TEM-1B_ gene with a unit transposon Tn2 and additional resistance elements and chromosomal point mutations such as *flo*R, *gyr*A, *par*C, *sul*2, *tet*(A), *tet*(B), and *tet*(M) (Table S3). The remaining AmpC isolates were classified as ST162 (n = 3), ST306 (n = 1), and ST20 (n = 1). A plasmidal AmpC gene was found located in insertion sequence ISEc9 and unit transposon Tn6196 in ST20 isolate contig whereas a chromosomally encoded AmpC gene was found in IS26 in a single ST306 isolate. The distribution of β-lactamase genes predicted the origin of the contigs, and contig-related data along with the source isolate information are presented in Table S2. A list of additional genes and their conferred phenotypic resistance, along with the plasmidal distributions, are provided in Table S3.

## 4. Discussion

We conducted a double-blind, randomized controlled feedlot trial that measured the effects of extended-spectrum β-lactam and macrolide antibiotics on cattle fecal *E. coli* populations by focusing on extended-spectrum cephalosporin-resistant *E. coli* in feces collected both before (Day 0) and after antibiotic treatment (Days 7, 14, 28, 56) up until slaughter age (Day 99). According to our results, following ceftiofur treatment a significant increase in the proportion of 3GC resistant *E. coli* was observed concomitant with an overall reduction in total *E. coli* in cattle feces. However, these effects were only observed in cattle receiving ceftiofur and these emerged immediately after antibiotic treatment and through Day 7 post-treatment. The *E. coli* population recovered to baseline levels by Day 14 and remained the same until slaughter (Figure 1 and Figure 2).

Our results confirmed the findings of a 28-day-long cohort study conducted by Lowrance et al. (2007) that showed an increase of ceftiofur-resistant *E. coli* in cattle feces following variable dosages of ceftiofur, most of which recovered to initial levels by 14 days to 28 days [26]. Our findings also matched with another 26-day-long randomized controlled study (Kanwar et al.) that revealed that use of ceftiofur significantly increased (*p* < 0.05) the probability of recovering ceftiofur and multidrug-resistant *E. coli* 4 days after treatment; however, this was not significant (*p* > 0.05) from Day 12 until the end of the study [27,28]. A similar transient effect of ceftiofur on *E. coli* was also observed in the fecal microbiota of beef cattle [30].

According to a follow-up study by Alali et al. (2009) using the fecal samples from Lowrance, there was an increase in the number of *bla*_CMY-2_ genes immediately after the ceftiofur treatment (by Day 6) and a reduction in the 16srRNA gene numbers in the fecal microbiota compared to the control cattle [29]. Similar trends were also observed in another study by Kanwar et al. focusing on *bla*_CMY-2_ and *bla*_CTX-M_ genes in the fecal microbiota of beef cattle up to 26 days after ceftiofur treatment [27]. Both studies reported recovery of gene copy numbers to baseline levels by Day 14. These results were also supported by a recent microbiome study published by Weinroth et al., (2018), which showed no difference in the β-lactamase genes in the fecal microbiome of beef cattle obtained from feces collected before (Day 0) and after ceftiofur treatment (Day 26) [30]. Based on such evidence, potential public health risks related to the selection of extended-spectrum β-lactam resistant microorganisms in cattle appear to be heavily dependent on the time between the drug administration and slaughter. According to Federal regulations (21CFR522, Section 522.313a) in the United States, a 13-day slaughter withholding period is required after the last ceftiofur crystalline-free acid treatment. When time-dependent effects of this ceftiofur formulation on the fecal AMR population of cattle are considered, this withholding period should be sufficient to reduce AMR pathogens at slaughter to baseline levels. However, such dynamics are also heavily dependent on the distribution of the pre-existing bacterial population, their AMR profiles, and their plasmidal presence and distribution that are influenced by previous and ongoing farm management strategies, environment, and the historical and concurrent uses of other antibiotics.

Farm environment was also shown as one of the potential drivers of cephalosporin-resistant *E. coli* populations that are shown to be varied by individual farms, regardless of the ongoing use of ceftiofur and other antibiotics [32,42,43,44]. Historical use of antibiotics may also have selected for antimicrobial resistance–and 3GC-resistance specifically–in the farm environment, since previous lots of cattle receiving ceftiofur will have metabolized and then excreted ceftiofur through their urine. This, in turn, may potentially contribute to further selection of extended-spectrum cephalosporin-resistant microorganisms in the environment [45,46]. This could perhaps explain the findings of one study that revealed higher 3GC resistant *E. coli* prevalence on hides when compared to the feces of cattle receiving ceftiofur [47].

In our study, certain genotypic and phenotypic AMR profiles of *E. coli* isolates that were identified in the same 7-gene MLST group were almost identical at pen- and animal-level (Tables S2 and S3). Similarly, a clonal *Salmonella* population in the same group of cattle was previously reported to be clustered by animal housing (pens) and cattle sources [48]. One of the limitations of our study was in exploring the genotypic features of only a subset of purposively chosen ESBL/AmpC *E. coli*; therefore, the distribution of clonal *E. coli* in cattle and other related patterns remains unknown.

The contribution of the use of other cattle antibiotic classes in the co-selection of ESBL/AmpC *E. coli* has been shown previously [49,50]. For example, the use of chlortetracycline was shown to play a critical role in the selection and maintenance of ceftiofur-resistant *E. coli* levels due to *bla*_CMY-2_ and *tet(A)* gene being carried on the same plasmid [28]. In contrast, another study showed no effect of chlortetracycline exposure on tetracycline and ceftiofur co-resistant *E. coli* in beef cattle [51]. Our study was the first that measured the effects of the macrolide tulathromycin on ESBL/AmpC *E. coli* and we did not observe co-selection of β-lactam resistance with the use of this drug. This was perhaps not entirely expected given that ceftiofur and tulathromycin-resistant *E. coli* were recovered both before and after treatment, along with the isolates that carried both *bla*_CTX-M_ and *mph*(A) genes in the same presumptive plasmid contig (Table S2). However, using the same logical thinking the use of other antibiotics such as aminoglycosides, tetracyclines, and fluoroquinolones could likewise enhance the ESBL/AmpC *E. coli* population in the same cattle involved in our study. This was not measured since these treatments were not applied.

In our study, we identified Inc class plasmids that have been shown to transfer ESBL and AmpC resistance elements between *Salmonella* and *E. coli* [52]. However, when our results are compared with previous research findings that revealed the majority of *Salmonella* isolates recovered from the same research cattle were pan-susceptible, regardless of ceftiofur and tulathromycin treatment, this was not the case [48,53]. Based on the current literature, the IncA/C plasmid is one of the major plasmids that harbors *bla*_CMY-2_ while *bla*_CTX-M-32_ genes were previously found in IncN plasmids of beef cattle origin *E. coli* [54,55]. The Inc A/C plasmid was also shown to be highly stable under the selection pressure of ceftiofur [56]. Although we identified an IncA/C plasmid and *bla*_CMY-2_ gene from a single control steer in our study, the majority of plasmidal origin *bla*_CMY-2_ genes were found on an IncX4 plasmid along with the ISEc9 element. This was isolated from the same ceftiofur treated steer both before and after ceftiofur treatment, and repeatedly over 3 sampling periods until Day 56. In our study, we did not identify any IncN plasmids, and *bla*_CTX-M-2_ genes were mainly predicted to be on the chromosome along with ISKpn26. However, due to the small sample size of isolates that were explored using WGS, such findings cannot be generalized to the *E. coli* population of our study. Although it would have been ideal to whole-genome sequence all of the isolates collected for this study, budget restrictions meant that only a random subset of isolates from each sampling day and treatment with different ESBL and/or AmpC resistance phenotypes was subjected to WGS analysis. This limited our ability to learn about the cattle population’s genetic AMR profiles and MGEs throughout the entire feeding period.

Beyond higher generation cephalosporins, carbapenems are one of the last human-reserved β-lactam antibiotics that can treat infections caused by the ESBL/AmpC-producing bacteria. Even though carbapenems are not permitted for use in food-producing animals in the United States, carbapenem-resistant bacteria have been found in livestock [57,58]. Recent evidence suggests the use of older-generation beta-lactam antibiotics in livestock can select for carbapenem-resistant *E. coli* [59] which makes this an urgent public health threat to be considered when choosing antibiotics for disease control in food-producing animals [9].

Questions also sometimes arise concerning defining injudicious, overuse or misuse of antibiotics. Concerning the two classes and formulations of drugs studied in this project (i.e., a third-generation cephalosporin formulated as ceftiofur crystalline-free acid and a macrolide in the form of tulathromycin), both products require a veterinary prescription and since 2012 the ceftiofur product generally cannot be used in any extra-label manner in the United States, especially in cattle. A recent paper by Hope et al. (2020) documents the quantitative use of these two drugs in cattle feedlots; specifically, for two major labeled indications for bovine respiratory disease (BRD) as treatment and control (or, metaphylaxis [60]). On a strictly bulk basis (e.g., mass in grams) relatively few of these are used when compared to, for example, tetracyclines or ionophores. However, on a per regimen-administered basis these are used relatively frequently with cephalosporins administered to 0.112 of each animal-year (AY) for BRD pen-level control and macrolides were administered to 0.265 AY in 2016. In terms of treatment of individual animal cases of BRD, these same two drugs/classes were administered to 0.028 and 0.08 regimens per AY. In another paper (Dedonder et al., 2015) the number needed to treat (NNT) for cattle respiratory disease when used as therapy was estimated using forest-plots from meta-analyses as being approximately 6 (i.e., six animals are treated for every one animal saved from mortality) and it is 5 for control uses (i.e., five animals received metaphylaxis (control) for every single animal cured or saved from later morbidity) [61].

## 5. Conclusions

In conclusion, though our results showed no long-term effects of ceftiofur and tulathromycin treatments on the AmpC/ESBL fecal *E. coli* population of beef cattle, when the observed number and variety of MDR profiles in the same *E. coli* are considered, any class of medically important antibiotics should be used with judicious care given the great potential that exists for co-selection by other antibiotics.

There were temporary and measurable effects of ceftiofur use on fecal *E. coli* populations in the 7 days following treatment, which has previously been documented by our group and others. By slaughter age, any differences between the two antibiotic treated groups and the control group had disappeared which has not previously been reported in any longitudinal studies. Disconcertingly, we found evidence that genes encoding for resistance to three of the five highest priority critically important antimicrobials (i.e., 3rd generation cephalosporins, macrolides, and fluoroquinolones) per the World Health Organization) are appearing within single *E. coli* strains. This suggest that the use of any of these classes of antimicrobial could expand the prevalence of all of them should these strains increase in number.

## Figures and Tables

**Table 1:** Proportion of AmpC, ESBL, and ESBL/AmpC phenotypes across treatments and days.

	Day 0		Day 7		Day 14		Day 28		Day 56		Day 99	
	AmpC	ESBL	AmpC	ESBL	AmpC	ESBL	AmpC	ESBL	AmpC	ESBL	AmpC	ESBL
**Ceftiofur**	0.10 (4/40)	0.00 (0/40)	0.13 (5/40)	0.15 (6/40)	0.13 (5/40)	0.05 (2/40)	0.08 (3/40)	0.05 (2/40)	0.21 (8/39)	0.15 (6/39)	0.18 (7/39)	0.00 (0/39)
ESBL/AmpC	0.10 (4/40)		0.28 (11/40)		0.18 (7/40)		0.13 (5/40)		0.36 (14/39)		0.18 (7/39)	
**Control**	0.04 (2/53)	0.09 (5/53)	0.02 (1/54)	0.04 (2/54)	0.13 (7/54)	0.02 (1/54)	0.11 (6/54)	0.00 (0/54)	0.19 (10/54)	0.02 (1/54)	0.16 (9/54)	0.02 (1/54)
ESBL/AmpC	0.13 (7/53)		0.06 (3/54)		0.15 (8/54)		0.11 (6/54)		0.22 (12/54) *		0.19 (10/54)	
**Tulathromycin**	0.20 (8/40)	0.08 (3/40)	0.13 (5/40)	0.03 (1/40)	0.23 (9/40)	0.03 (1/40)	0.13 (5/40)	0.13 (5/40)	0.00 (0/39)	0.08 (3/39)	0.18 (7/39)	0.03 (1/39)
ESBL/AmpC	0.28 (11/40)		0.15 (6/40)		0.25 (10/40)		0.25 (10/40)		0.08 (3/39)		0.21 (8/39)	

* Presence of a single isolate that is not classified as either AmpC or ESBL phenotype.

## Data Availability

Full sequencing data from this project can be found under NCBI BioProject No. PRJNA625741.

## Supplementary Materials

The following supporting information can be downloaded at: https://www.mdpi.com/article/10.3390/microorganisms10102071/s1, Table S1. Descriptive statistics related to log10 CFU counts obtained from MAC, MCef plates and their differences (log10 MCef–MAC) presented by day and individual treatment groups. Table S2. Mobile genetic elements, antibiotic resistance genes, and plasmids defined in the contigs carrying a *bla* gene and their quality metrics and origin of predictions by 7-gene MLST, day, pen, and individual animals. Table S3. Genotypic and phenotypic AMR resistance and plasmidal distributions of ESBL and AmpC phenotype *E. coli* recovered from the study.
