# Supplementary material for: Long-Term Effects of Single-Dose Cephalosporin or Macrolide Use on the Prevalence of AmpC and Extended-Spectrum β-Lactamase Producing Escherichia coli in the Feces of Beef Cattle
Source: Microorganisms. 2022 Oct 20;10(10):2071. doi: 10.3390/microorganisms10102071 (PMC9610231; doi:10.3390/microorganisms10102071)
Supplement: Supplementary file 1 [file microorganisms-10-02071-s001.zip › microorganisms-1971775-supplementary.pdf]

		MAC						MCef						Difference (Mcef-MAC)					
	Days	0	7	14	28	56	99	0	7	14	28	56	99	0	7	14	28	56	99
Control	Obs.	53	54	54	54	54	53	53	54	54	54	54	54	53	54	54	54	54	54
	Mean	6.53	5.31	5.20	5.96	6.01	6.55	0.34	0.20	0.45	0.19	0.20	1.76	-6.20	-5.12	-4.79	-5.77	-5.81	-4.67
	Median	6.60	5.67	5.34	5.91	6.13	6.64	0.00	0.00	0.00	0.00	0.00	0.00	-6.57	-5.63	-5.30	-5.87	-6.10	-5.82
	Min	4.92	0.00	0.00	4.75	0.00	4.81	0.00	0.00	0.00	0.00	0.00	0.00	-7.49	-7.43	-7.47	-7.33	-7.53	-7.38
	Max	7.49	7.43	7.47	7.33	7.53	7.58	6.49	5.41	6.84	6.19	5.72	7.28	0.01	0.50	3.60	-0.44	5.26	0.00
Tulathromycin	Obs.	40	40	40	40	38	39	40	40	40	40	39	39	40	40	40	40	39	39
	Mean	6.48	5.60	5.43	6.19	6.42	6.63	0.58	0.11	0.12	0.61	0.15	1.05	-5.90	-5.49	-5.30	-5.58	-6.25	-5.58
	Median	6.84	5.63	5.49	6.33	6.55	6.71	0.00	0.00	0.00	0.00	0.00	0.00	-6.81	-5.51	-5.49	-6.16	-6.44	-6.34
	Min	0.00	0.00	0.00	4.60	4.08	4.88	0.00	0.00	0.00	0.00	0.00	0.00	-7.59	-7.58	-7.34	-7.50	-7.56	-7.31
	Max	7.59	7.58	7.34	7.52	7.56	7.54	6.78	4.30	4.96	5.58	5.78	5.26	4.60	0.00	0.00	-0.07	-1.70	-1.05
Ceftiofur	Obs.	40	40	40	39	39	38	40	40	40	40	39	38	40	40	40	40	39	38
	Mean	6.56	1.89	5.19	6.20	6.30	6.70	0.52	0.40	1.11	0.95	0.71	1.38	-6.03	-1.49	-4.08	-5.09	-5.59	-5.13
	Median	6.98	0.00	5.30	6.27	6.28	6.64	0.00	0.00	0.00	0.00	0.00	0.00	-6.81	0.00	-4.68	-6.06	-6.07	-6.33
	Min	3.90	0.00	0.00	4.83	5.17	5.64	0.00	0.00	0.00	0.00	0.00	0.00	-7.58	-5.60	-6.99	-7.53	-7.62	-7.51
	Max	7.58	5.60	6.99	7.53	7.62	7.58	6.27	4.94	5.55	5.71	5.53	6.82	-0.30	3.90	0.35	0.00	-0.98	0.00

Table S1. Descriptive statistics related to log10 CFU counts obtained from MAC, MCef plates and their differences (log10 MCef–MAC) presented by day and individual treatment groups.

Gene (NCBI GeneBank #)	Day	Pen	Animal ID	7-gene MLST	Contig			Posterior probability		Prediction result	Mobile element	Plasmid and antibiotic resistance genes
					ID	Length	Cov[a]	Chrom[b]	Plasmid			
blaCMY-2_1 (X91840)	0	9	4243	156	32	271,916	26.0	0.121	0.879	Plasmid	ISEc9	IncX4_2
	28	9	4243	156	34	37,036	52.8	0.121	0.879	Plasmid	ISEc9	IncX4_2
	7	9	4243	156	46	37,036	30.8	0.195	0.805	Plasmid	ISEc9	IncX4_2
	99	9	4249	20	19	100,155	34.0	0.127	0.873	Plasmid	ISEc9, Tn6196	aadA15_1, aadA2_1, ant(2'')-Ia_1, aph(3'')-Ib_5, aph(6)-Id_1, cmlA1_1, floR_2, sul1_5, sul2_2, tet(A)_6
	0	51	4300	162	97	2,275	152.7	0.111	0.889	Plasmid	-	-
	7	53	4337	162	33	48,375	37.7	0.759	0.241	Chromosome	IS26	-
	0	55	4274	162	67	2,263	197.0	0.641	0.359	Chromosome	-	-
blaCTX-M-1_1 (DQ915955)	99	54	4350	744	72	3,155	117.1	0.845	0.155	Chromosome	-	-
	28	57	4315	306	109	3,966	60.1	0.584	0.416	Chromosome	-	mph(A)
	28	57	4329	306	124	3,966	63.7	0.584	0.416	Chromosome	-	mph(A)
	28	58	4330	8443	153	15,086	53.0	0.248	0.752	Plasmid	-	mph(A)
	0	59	4318	8443	128	15,086	27.1	0.248	0.752	Plasmid	-	mph(A)
blaCTX-M-27 (HQ398215)	56	56	4290	17	90	3,967	24.9	0.559	0.441	Chromosome	IS102	-
	56	58	4330	17	106	3,967	38.2	0.340	0.660	Plasmid	IS102	-
blaCTX-M-32_2 (AJ557142)	56	9	4234	11460	132	5,626	17.2	0.786	0.214	Chromosome	-	-
	0	54	4263	224	55	4,906	19.3	0.748	0.252	Chromosome	ISKpn26	-
	0	56	4295	224	39	4,906	22.9	0.748	0.252	Chromosome	ISKpn26	-
	14	56	4295	224	50	4,906	26.2	0.841	0.159	Chromosome	ISKpn26	-
	0	58	4268	224	31	4,906	46.3	0.748	0.252	Chromosome	ISKpn26	-
blaCTX-M-55_1 (DQ810789)	99	56	4339	1725	29	35,378	21.4	0.119	0.881	Plasmid	ISEc9, IS629	IncFIC(FII)_1
blaTEM-1A_1 (HM749966)	99	54	4350	744	55	8,617	214.4	0.126	0.874	Plasmid	-	aph(3'')-Ib_5, aph(6)-Id_1, sul2_2, tet(A)_6
	0	54	4263	224	49	13,748	23.0	0.540	0.460	Chromosome	ISVsa3	aph(3'')-Ib_5, aph(6)-Id_1, floR_2, sul2_2, tet(A)_6
	0	56	4295	224	33	13,748	27.5	0.540	0.460	Chromosome	ISVsa3	aph(3'')-Ib_5, aph(6)-Id_1, floR_2, sul2_2, tet(A)_6
	14	56	4295	224	40	13,748	35.8	0.022	0.978	Plasmid	ISVsa3	aph(3'')-Ib_5, aph(6)-Id_1, floR_2, sul2_2, tet(A)_6
	0	58	4268	224	28	13,748	43.7	0.022	0.978	Plasmid	ISVsa3	aph(3'')-Ib_5, aph(6)-Id_1, floR_2, sul2_2, tet(A)_6
	28	58	4330	8443	84	3,965	54.5	0.029	0.971	Plasmid	ISVsa3	aph(3'')-Ib_5, aph(6)-Id_1, floR_2, sul2_2, tet(A)_6
	0	59	4318	8443	79	3,965	27.7	0.029	0.971	Plasmid	ISVsa3	aph(3'')-Ib_5, aph(6)-Id_1, floR_2, sul2_2, tet(A)_6
blaTEM-1B_1 (AY458016)	0	9	4243	156	4	37,035	105.7	0.979	0.021	Chromosome	Tn2	-
	28	9	4243	156	3	271,916	31.6	0.979	0.021	Chromosome	Tn2	-
	7	9	4243	156	34	59,819	20.7	0.977	0.023	Chromosome	Tn2	-

[a] *coverage*

[b] *chromosome*

Table S2. Mobile genetic elements, antibiotic resistance genes, and plasmids defined in the contigs carrying a bla gene and their quality metrics and origin of predictions by 7-gene MLST, day, pen, and individual animals.

ESBL/AmpC phenotype	Day	Pen	ID	Trt[a]	7-gene MLST	AMR phenotype	AMR genotype[b]	Plasmid[c]
AmpC	99	9	4249	cont	20	Amoxicillin-clavulanic acid, Ampicillin, Ceftiofur, Ceftriaxone, Chloramphenicol, Gentamicin, Streptomycin, Sulfisoxazole, Tetracycline	blaCMY-2, cmlA1, floR, sul1, sul2, tet(A), tetC, aadA15, aadA2, ant(2'')-Ia, aph(3'')-Ib, aph(6)-Id,	IncA/C2, IncFII
AmpC	0	9	4243	cef	156	Amoxicillin-clavulanic acid, Ampicillin, Cefoxitin, Ceftiofur, Ceftriaxone, Chloramphenicol, Ciprofloxacin, Nalidixic acid, Sulfisoxazole, Tetracycline	blaCMY-2, blaTEM-1, floR, gyrA, parC, sul2, tet(A), tet(B), tet(M),	IncX4, IncY
AmpC	7	9	4243	cef	156	Amoxicillin-clavulanic acid, Ampicillin, Cefoxitin, Ceftiofur, Ceftriaxone, Chloramphenicol, Ciprofloxacin, Nalidixic acid, Streptomycin, Sulfisoxazole, Tetracycline	blaCMY-2, blaTEM-1, floR, gyrA, parC, sul2, tet(A), tet(B), tet(M),	IncX4, IncY
AmpC	28	9	4243	cef	156	Amoxicillin-clavulanic acid, Ampicillin, Cefoxitin, Ceftiofur, Ceftriaxone, Chloramphenicol, Ciprofloxacin, Nalidixic acid, Sulfisoxazole, Tetracycline	blaCMY-2, blaTEM-1, floR, gyrA, parC, sul2, tet(A), tet(B), tet(M),	IncX4, IncY
AmpC	0	55	4274	cef	162	Amoxicillin-clavulanic acid, Ampicillin, Cefoxitin, Ceftiofur, Ceftriaxone, Tetracycline	blaCMY-2, tet(A), tet(B), tet(M),	IncHI1B
AmpC	0	51	4300	tul	162	Amoxicillin-clavulanic acid, Ampicillin, Cefoxitin, Ceftiofur, Ceftriaxone, Tetracycline	blaCMY-2, tet(A), tet(B), tet(M)	IncHI1B
AmpC	7	53	4337	cont	162	Amoxicillin-clavulanic acid, Ampicillin, Cefoxitin, Ceftiofur, Ceftriaxone, Tetracycline	blaCMY-2, tet(A), tet(B), tet(M),	IncHI1B
AmpC	28	57	4329	cef	306	Amoxicillin-clavulanic acid, Ampicillin, Cefoxitin, Ceftiofur, Ceftriaxone, Chloramphenicol, Ciprofloxacin*, Streptomycin, Sulfisoxazole, Tetracycline	blaCTX-M-1, floR, qnrB19, sul2, tet(A), aph(3'')-Ib, aph(6)-Id, mph(A)	IncFII, IncR, IncX1
ESBL	56	56	4290	tul	17	Ampicillin, Ceftiofur, Ceftriaxone	blaCTX-M-27	p0111, IncB/O/K/Z, IncFII
ESBL	56	58	4330	tul	17	Ampicillin, Ceftiofur, Ceftriaxone	blaCTX-M-27	p0111, IncB/O/K/Z, IncFII
ESBL	0	54	4263	cont	224	Ampicillin, Ceftiofur, Ceftriaxone, Chloramphenicol, Ciprofloxacin, Nalidixic acid, Sulfisoxazole, Tetracycline	blaCTX-M-32, blaTEM-1, floR, gyrA, parC, parE, sul2, tet(A), aph(3'')-Ib, aph(6)-Id	IncFIC(FII), IncY
ESBL	0	58	4268	cont	224	Ampicillin, Ceftiofur, Ceftriaxone, Chloramphenicol, Ciprofloxacin, Nalidixic acid, Sulfisoxazole, Tetracycline	blaCTX-M-32, blaTEM-1, floR, gyrA, parC, parE, sul2, tet(A), aph(3'')-Ib, aph(6)-Id	IncFIC(FII), IncY
ESBL	0	56	4295	cont	224	Ampicillin, Ceftiofur, Ceftriaxone, Chloramphenicol, Ciprofloxacin, Nalidixic acid, Sulfisoxazole, Tetracycline	blaCTX-M-32, blaTEM-1, floR, gyrA, parC, parE, sul2, tet(A), aph(3'')-Ib, aph(6)-Id	IncFIC(FII), IncY
ESBL	14	56	4295	cont	224	Ampicillin, Ceftiofur, Ceftriaxone, Chloramphenicol, Ciprofloxacin, Nalidixic acid, Sulfisoxazole, Tetracycline	blaCTX-M-32, blaTEM-1, floR, gyrA, parC, parE, sul2, tet(A), aph(3'')-Ib, aph(6)-Id	IncFIC(FII), IncY
ESBL	28	57	4315	cef	306	Ampicillin, Ceftiofur, Ceftriaxone, Chloramphenicol, Ciprofloxacin, Streptomycin, Sulfisoxazole, Tetracycline	blaCTX-M-1, floR, qnrB19, sul2, tet(A), aph(3'')-Ib, aph(6)-Id,, mph(A)	IncFII(pSE11), IncR, IncX1
ESBL	99	54	4350	cont	744	Ampicillin, Ceftiofur, Ceftriaxone, Azithromycin, Chloramphenicol, Ciprofloxacin, Nalidixic acid, Streptomycin, Sulfisoxazole, Tetracycline, Trimethoprim-sulfamethoxazole	blaCTX-M-1, blaTEM-1, catA1, floR, gyrA, parC, sul1, sul2, tet(A), tet(B), aadA5, aph(3')-Ia, aph(3'')-Ib, aph(6)-Id, dfrA17, mph(A)	IncR
ESBL	99	56	4339	tul	1725	Ampicillin, Ceftiofur, Ceftriaxone, Chloramphenicol, Sulfisoxazole, Tetracycline	blaCTX-M-55, floR, sul3, tet(B), aph(3')-Ia	IncFIB
ESBL	0	59	4318	cont	8443	Ampicillin, Ceftiofur, Ceftriaxone, Chloramphenicol, Ciprofloxacin*, Streptomycin, Sulfisoxazole, Tetracycline	blaCTX-M-1, blaTEM-1, floR, qnrB19, sul2, tet(A), aph(3'')-Ib, aph(6)-Id, , mph(A)	IncR
ESBL	28	58	4330	tul	8443	Ampicillin, Ceftiofur, Ceftriaxone, Chloramphenicol, Ciprofloxacin*, Sulfisoxazole, Tetracycline	blaCTX-M-1, blaTEM-1, floR, qnrB19, sul2, tet(A), aph(3'')-Ib, aph(6)-Id, mph(A)	IncR
ESBL	56	9	4234	cef	11460	Ampicillin, Ceftiofur, Ceftriaxone	blaCTX-M-32, parC	IncFII(pSE11)

[a] Treatments: cef, ceftiofur; cont, control; tul, tulathromycin

[b] Point mutations are screened with %90 ID and 60% coverage and acquired resistance genes are screened with 97% ID and 97% coverage thresholds

[c] Plasmids are screened with 97% ID and 97% coverage thresholds

Table S3. Genotypic and phenotypic AMR resistance and plasmidal distributions of ESBL and AmpC phenotype E. coli recovered from the study.
